# A new twist to V-ATPases and cancer

**DOI:** 10.18632/oncotarget.25883

**Published:** 2018-08-07

**Authors:** Michael Forgac

**Affiliations:** Michael Forgac: Department of Developmental, Molecular and Chemical Biology, Tufts University School of Medicine, Boston, MA, USA

**Keywords:** V-ATPase, prostate cancer, androgen receptor, HIF-1 alpha, transferrin

V-ATPases are ATP-dependent proton pumps that both acidify intracellular compartments (such as lysosomes and secretory vesicles) as well as pump protons across the plasma membrane in mammalian cells [1]. They have been implicated in cancer growth and metastasis in a variety of contexts [2]. For example, isoforms of the a subunit that target V-ATPases to the cell surface (such as a3) have been shown to be important in the migration and invasion of breast cancer cells [3, 4] and to be overexpressed in highly invasive breast cancer cells and metastatic tumors [5]. In this context, they have been suggested to promote the activity of secreted, acid-dependent proteases that degrade the extracellular matrix, thus promoting tumor cell invasion. Inhibition of V-ATPases has also been shown to lead to apoptosis of tumor cells [6], which appear to be more highly sensitive to V-ATPase inhibition than normal cells. This increased sensitivity may stem from the fact that many tumor cells have an enhanced reliance on glycolytic metabolism, resulting in a higher-than-normal load of cytosolic acid. V-ATPases have also been shown to play an important role in a variety of cell signaling pathways, including Wnt, Notch, mTORC1 and AMPK [2]. Because of the importance of these pathways in tumor cell progression, V-ATPases represent an important signaling node in cancer cells. Finally, V-ATPases have been implicated in the drug resistance of tumor cells [2].

In the current paper by Licon-Munoz, et al, [7] a new mechanism by which V-ATPase inhibition can be used to target prostate cancer cells is described. Prostate cancer is a leading cause of death among men and new treatments for this disease are needed. Prostate cancer cells (as well as normal prostate cells) express androgen receptor (AR) and ablation of AR has been shown to be effective in the treatment of androgen-dependent prostate tumors. AR expression is controlled, in part, by the transcription factor hypoxia inducible factor 1-alpha (HIF-1α), which suppresses the expression of AR. Normally, HIF-1α is hydroxylated by prolyl hydroylases that result in its proteosomal degradation. Inhibiting these prolyl hydroxylases would therefore result in stabilization of HIF-1α, leading to decreased expression of AR. Prolyl hydroylases are iron-dependent enzymes which, therefore, depend upon the normal delivery of iron to cells via the transferrin pathway. Transferrin is taken up by cells via receptor-mediated endocytosis and, upon exposure to low pH within the endosome, releases its iron. Iron is then transported into the cytosol, where it can be used to support iron-dependent enzymes, including the prolyl hydroxylases.

The current study shows that, by treating prostate cancer cells with a specific inhibitor of V-ATPases (concanamycin), the pH of the endosomal compartment is increased, resulting in defective iron delivery to cells by the transferrin system. This iron deficiency results in increased HIF-1α levels, most likely due to decreased prolyl hydroxylase activity, and a corresponding decrease in AR levels. These results help explain their previous report that V-ATPase inhibition results in decreased levels of mRNA for prostate-specific antigen (PSA), a gene whose expression is controlled by AR [8]. The mechanism proposed by Licon-Munoz et al. [7] is supported by a number of lines of evidence. This evidence includes the decreased AR levels and increased HIF-1α levels observed in cells treated with a prolyl hydroxylase inhibitor and the ability to reverse the effects of concanamycin treatment on AR and HIF-1α levels by treatment of cells with supplemental iron.

While concanamycin inhibits the V-ATPase in all cells, not just tumor cells, the reported findings open up a new avenue for the control of AR expression in prostate cancer cells, with the prospect of tumor-specific inhibition of V-ATPases an attractive therapeutic avenue for the treatment of a variety of cancers. In this regard, the possibility of identifying isoforms of V-ATPase subunits that are particularly important for tumor cell survival and metastasis represents an exciting and novel approach in the development of anti-cancer drugs [2, 5]. Moreover, although changes in gene expression as a result of changes in V-ATPase activity in cancer cells have previously been reported [9], the newly reported mechanism linking V-ATPase activity and transcriptional control is novel and represents a possible approach to the control of gene expression in cancer. One intriguing possibility is that the transcriptional regulation of hormone receptors may prove to be a useful target in the treatment of other hormone-responsive cancers.
